# The practical use of Kit-Build concept map on formative assessment

**DOI:** 10.1186/s41039-017-0060-x

**Published:** 2017-09-22

**Authors:** Jaruwat Pailai, Warunya Wunnasri, Kan Yoshida, Yusuke Hayashi, Tsukasa Hirashima

**Affiliations:** Graduate School of Engineering, Hiroshima University, 1-4-1 Kagamiyama, Higashi-Hiroshima City, 739-8527 Japan

**Keywords:** Formative assessment, Kit-Build concept map, Automatic concept map assessment, Lecture class

## Abstract

Formative assessment can encourage an instructor to improve learning achievements in a lecture class. The goal of formative assessment in a classroom situation is monitoring learners to provide instructor’s feedback for improving learner’s understanding as well as instructor’s expectation. A Kit-Build concept map is a digital tool for supporting a concept map strategy to represent instructor’s expectation and to assess the current understanding of learners. The Kit-Build concept map is also adequate for implementing the formative assessment in a lecture class. The proposition level exact matching between the concept map of instructor and learners can generate the diagnosis results for informing the instructor about the gaps between current learner’s understanding and the instructor’s expectation. Accordingly, the instructor can design the feedback based on the diagnosis results for improving the understanding of learners. In this paper, we propose the arrangement of the ability of the Kit-Build concept map on formative assessment in a lecture class for creating an opportunity to assess current understanding of learners as more as possible. And we present the effectiveness of the Kit-Build concept map on the closed-end approach in elementary school through three practical uses in various lecture classes, which illustrate the contribution of the Kit-Build concept map when utilized on formative assessment in the lecture class.

## Introduction

The formative assessment is a process which is used by instructors and learners during instruction. It provides feedback to adjust ongoing teaching and learning to improve learners’ achievement of intended instructional outcomes (Melmer et al., 2008). For implementing the formative assessment in lecture class, there is a series of key questions on formative assessment that we will mention as the requirement of formative assessment which are as follows: “Where are learners going?”, “Where are learners now?” and “How to close the gap?” (Moss & Brookhart, 2010). The information through formative assessment can encourage the instructor for giving the feedback to improve the understanding in a timely manner, which is the most efficient feedback (Wiliam et al., 2004). Also, the interaction based on formative information is the formative assessment key feature (Ballantyne et al., 2002). Accordingly, gathering and assessing the learning evidence for providing the feedback in a class period are the processes of completing the formative assessment, and are also creating an opportunity for improving learning achievements concurrently. Nevertheless, the effective implementation of formative assessment is problematic for an instructor on observing and interpreting the learning evidence in a class period. The instructor should recognize the current learning situation clearly before deciding the ways for improving the learners’ understanding. Particularly, it is difficult to identify the current common understanding and misunderstanding of learners when the instructor duels with a large number of learners in the lecture class. Hence, the essential characteristic of formative strategy not only elicits the current learning situation but also visualizes the observing information in an easily understandable form. Also, the technology-enhanced learning produces an accessing ability which can provide the information whenever the instructor needs to know the current learning situation.

The Kit-Build concept map is a digital tool for supporting a concept map strategy. The ability of the Kit-Build concept map includes a construction tool where users can construct concept maps, and an automatic concept map assessment where the system can report diagnosis results (Hirashima et al., 2015). In this paper, the Kit-Build concept map places a strong emphasis on implementing formative assessment in a lecture class. We propose an arrangement of the Kit-Build concept map on formative assessment. The main contribution of the Kit-Build concept map on formative assessment in a lecture class is creating, gathering, and assessing the evidence of learners to generate instant practical information for designing and providing instructor’s feedback. The proposition level exact matching methodology is an automatic assessment of the Kit-Build concept map. The diagnosis results of propositional exact matching can inform the instructor immediately on the current understanding of learners and also when learners understand the lecture content differently from the instructor’s expectation. The diagnosis results are a confirmation of the understanding between an instructor and learners on lecture contents. Especially, the group-diagnosis results can inform overview of class on only one map, which is the common understanding and misunderstanding based on the assessment results of learner’s evidence. Accordingly, we present the practical use in the classroom situation when the Kit-Build concept map is utilized on three lecture classes which are implemented by formative assessment. And the results of practical uses represent the effectiveness of the Kit-Build concept map on formative assessment.

## Background

The formative assessment approach is used to monitor learning of learners for providing ongoing instructor’s feedback, which is a key for helping the learners to achieve a learning goal. Also, monitoring is assessing learner’s evidence of class and for examining the learner’s knowledge via the formative assessment strategy. The selected strategy is used to illustrate both of the learning goal and the evidence of learners for determining a learning gap. An appropriate strategy should present an expectation of the instructor as well as the “where are learners going?” obviously, and also should represent the understanding of learners as more as possible for identifying the “where are learners now?” clearly.

A lecture class is an educational talk of an instructor for sharing knowledge to learners, while the instructor expects learners to understand the lecture contents positively. The instructor is an expert of lecture contents who has the content expertise and can use his/her experience to raise the understanding of learners, while the learners are the participant of knowledge sharing, who are a creator of evidence to present their understanding on what they can grasp and perceive following the lecture. The evidence of learners can represent the current learning situation in the class, which can be used to determine the gaps in learning when comparing against the learning goal of the class. Thus, the results of the comparison can indicate the learning achievements when learners reach the learning goal and also indicate the learning gaps when learners struggled to understand the lecture. The gaps are critical areas of the class which require the supplementary explanation of the instructor to improve the learner’s understanding as well as answering of “How to close the gap?”

For applying a strategy of formative assessment, it requires to create both a learning goal of class and evidence of learners. For instance, the perfect score of multiple choice questions is a learning goal of the class. Also, the learner’s evidence is the answer sheets, and difference scores can determine the gaps between the expectation of instructor and the understanding of learners. However, the characteristics of the proper strategy for implementing formative assessment should represent the understanding of learners as more as possible. Concept maps become to be the proper formative assessment strategy because its characteristics can be a response to formative assessment strategy’s requirement which can adequately represent the expectation of an instructor and the understanding of learners clearly.

### Concept map strategy

Concept maps are graphical tools that are used to represent and organize knowledge (Novak & Cañas, 2008). A proposition of concept map is a unit of meaning, which is constructed by connecting two concepts via a relation with linking words. The propositions include concepts and relations that are a core component of measuring a map score. The traditional concept map assessment is evaluated by using criteria or via a human-based rubric. The principal point of each criterion depends on the objective of assessment. For instance, Novak’s assessment methodology emphasizes the hierarchy and crosslinks (Novak & Gowin, 1984). A correct proposition can get only one score, while for the specific propositions, which are the connection between two concepts from the different segments of the map, the score will be increased from one to ten. It indicates the characteristic of the crosslink. Moreover, five additional scores will be given for every correct hierarchy in a map. The other rubrics attend to graph structure like branching and grouping of propositions (Cronin et al., 1982) or continuous rating scales of linking words, sophisticated, and cooperation (Bartels, 1995; NCSEC, 2000; Mueller, 2007). In addition, the concerns of concept map assessment are quality and quantity of propositions, which are the general discussion when the assessment methods are proposed.

Concept map strategy is used in education areas to represent and assess knowledge of learners in classes. An instructor can gain the current learning information and then give the feedback based on the information in various situations. For instance, using concept maps on the individual or group discussion can contribute self-awareness of learners (Buldu & Buldu, 2010). An instructor can use concept maps as a formative strategy. The criteria map represents a learning goal of a class in a concrete form, which is used to compare with the concept map of learners to find discrepancies based on the criteria map before the instructor gives the feedback to the learners (Trumpower & Sarwar, 2010). Accordingly, several researchers presented that the concept map strategy is simple to use, effective, and satisfactory on problem-solving in classroom situations (Schacter et al., 1999; Hsieh & O’Neil 2002). The concept map is an effective strategy in a classroom situation that affects learners’ achievements and interests. Although the traditional lecture class contributed learning achievements and meaningful learning in the classroom situation, the concept map can significantly improve learning achievements of learners when compared with lecturing and is also more effective than lecturing in encouraging meaningful learning (Chularut and DeBacker, 2004; Chiou, 2008; Aghakhani et al., 2015).

The traditional concept map strategy is a useful strategy for representing knowledge, and its characteristics can respond to the requirement of formative assessment on where learners’ questions are going and also where learners’ questions are suitable now. Although the remaining requirement is how to close the gap question, the instructor should identify the gap before finding the way to close it. “What is the gap?” is an implicit question of how to close the gap question. Thus, the comparison results of the criteria map against the learner’s concept map can identify what the gap is based on the traditional concept map strategy. However, it is very difficult for the instructor to examine each concept map built by learners in the class in real time. So, using the traditional concept map as the formative strategy without technology enhancement is an important focused issue when it is implemented in classroom situation practically.

### Kit-Build concept map

The framework of the Kit-Build concept map is designed based on a concept map strategy, which includes a concept map construction tool, an automatic concept map assessment, and an analyzer of instructor. The significant component of the Kit-Build concept map is a “kit.” The kit consists of the concepts and the relations with linking words. These components are extracted from a concept map of an instructor (as we called “goal map”) on the segmentation task. An automatic assessment methodology of the Kit-Build concept map is a proposition level exact matching between a goal map and concept map of learners (as we called “learner map”). These abilities respond to the concerns of concept map assessment following a kit which is the quantity controller, and to the propositional exact matching based on the goal map which is the quality controller.

**Fig. 1:**
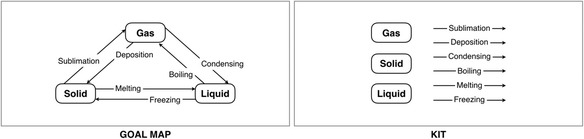
An example of the goal map and its kit

**Fig. 2:**
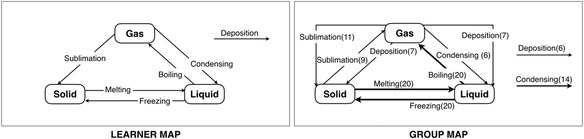
An example of the learner map and the group map

Figure 1 shows an example of a goal map and its kit. An example of learner map on “Change of State: Solid, Liquid, and Gas” is shown in Fig. 2, which is integrated from the kit on the structuring task. The learner map of the Kit-Build concept map is constructed by using only the components of the kit, which is different from the traditional concept maps where all of concepts and links are drawn by the learner. All of the learner map components are the same concepts and relations with the goal map, but the propositions can be possible to be different from the goal map. So, it is practicable to use the proposition level exact matching for indicating the difference between the goal map and the learner map directly. Moreover, the Kit-Build concept map can generate an additional evidence of learners as a group map for displaying the common understanding of all learners in the class (Fig. 2). The thickness line and a tagged number in parenthesis refer to the number of learners who connect those links. The weight of line represents the degree of learners which means the bolder line present the number of learners more than the other thin line, and also correspond to the tagged number of each link.

The proposition level exact matching is an assessment methodology of the Kit-Build concept map, which can implement as automatic assessment. The proposition level exact matching is the comparison of each proposition of learner maps against the goal map for identifying the similarity and difference of current understanding of learners and the instructor’s expectation. The analyzer can provide the diagnosis results that include similarity scores, a group map, and a difference map. A similarity score is percentages of each learner map when a learner map is compared with the goal map. The results can show achievements of learners based on the instructor’s expectation. Also, the difference map displays the mismatch of each learner map or the group map based on the goal map in the form of three types of error link, which include lacking links, excessive links, and leaving links. The link that is used to connect two concepts in the learner map but at least one concept which is different from the goal map is called excessive link. The link that is not connected to any concept is leaving links. And the lacking links are used to call the link that is in the goal map but does not exist in the learner map.

**Fig. 3:**
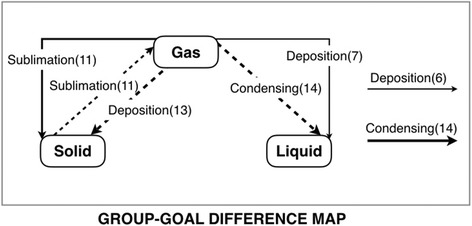
An example of the group-goal difference map

In the difference map, the concepts will be located same as the concepts in the goal map and only relations of mismatch propositions are displayed. An example of a group-goal difference map is shown in Fig. 3. The map displays three types of error links same as the individual-goal difference map. The excessive link is represented in the form of solid line which the link is connected with two concepts. It can identify the relations that have the confusion or the misunderstanding of learners, and the tagged number presents the number of learners who constructed the link. The leaving link is represented in the form of a solid line in which the link is not connected with any concept. This link indicates that the learners do not understand the linking word. Also, the tagged number means the number of learners who do not use the link to connect with any concept. The dashed line represents the lacking link which is an error correction for displaying the correcting information of excessive and leaving links. The tagged number of lacking link is the total number of excessive link and leaving link, which related to the weight of line. The more tagged number in each relation will represent with a thicker line. For instance, “Deposition (13)” dashed line is the lacking link while “Deposition (7)” is the excessive link, and “Deposition (6)” is the leaving link. Moreover, the diagnosis results of the Kit-Build concept map are divided into individual-diagnosis results and group-diagnosis results. The individual-diagnosis results include individual-goal similarity scores and individual-goal difference maps. The individual-goal similarity score represents the achievement of each learner. Also, the individual-goal difference map represents the mismatch propositions between each learner map based on the goal map.

**Fig. 4:**
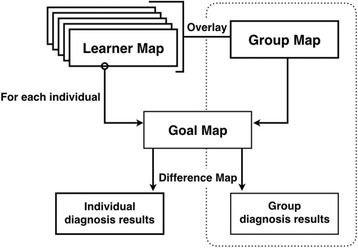
The analyzer workflow of the Kit-Build concept map

The group-diagnosis results include a group map, a group-goal similarity score, and a group-goal difference map. The group map displays the common understanding of learners on the lecture content, while the group-goal difference map displays the common misunderstanding of learners based on the instructor’s expectation. The filtering function of the Kit-Build analyzer can provide more efficient investigation by adjusting the intensity of three error types. The filtering function of group assessment is more explicit with the line weight. A thickness line and a number in parenthesis refer to the number of learners who connect those links. In addition, the link of each proposition is available for clicking to discover the learners who are the constructor of the link. Figure 4 illustrates the workflow of the analyzer when learners construct a map as a learner’s evidence. The learner maps will be evaluated through the propositional level exact matching methodology that is the procedure for reporting individual-diagnosis results. Also, the system can provide the additional procedure for reporting the group-diagnosis results at the same time.

Providing the components of the concept map is a kind of “closed-end” approach which is realizing the automatic diagnosis of the concept map built by a learner (Taricani & Clariana, 2006). The learner maps of the Kit-Build concept map are composed of the same components with the goal map. Hence, it is possible to detect the difference between them in the form of the diagnosis results. The learners are able to make a map in the limitation of providing parts, which is the difference from the traditional concept maps where learners can create concept map components by themselves. Therefore, the learners deal with only recall and understanding level in Bloom’s taxonomy (Bloom et al., 1956). Also, the components provided include concepts and links which are a middle of directedness of the mapping task and the score is an indicator of learner’s performance based on the possible maximum (Ruiz-Primo et al.,﻿ 2001; Ruiz-Primo, 2004). Thus, the components provided by the Kit-Build concept map can be used in the aspect of confirming the understanding between the instructor and learners in classroom situations with the benefit of the automatic assessment for implementing formative assessment.

In addition, several researches demonstrated the contribution of the Kit-Build concept map on learning effect (Alkhateeb et al., 2015, 2016a, 2016b; Funaoi et al., 2011). The contribution of the Kit-Build concept map framework has been researched in a reading comprehension topic where a direct interaction between the digital tool and learners has been examined. And the results show that Kit-Build concept map can help the learners retain and recall the information for a longer period of time. The provided concept map component illustrates effectiveness towards memory, same as the traditional concept map when the learning materials were the clear structure. In this paper, we emphasize the contribution of the formative assessment on learning effect which an instructor used the suggestion of the diagnosis results for improving learning achievements.

## Formative assessment in lecture class

The methodology of formative assessment is gathering and assessing the evidence of learning for designing and providing the instructor’s feedback, which improves learning achievements. Also, technology-enhanced learning can minimize the time of gathering the evidence through an assessing process, which is suitable for responding the time-limitation of a class period. It can inform the assessment results to the instructor in a short time that is necessary for implementing the formative assessment both inside and outside the classroom.

### Digital tools for supporting formative assessment

Reducing time consumption is an obvious reason to use digital tools. Storing and accessing the Internet are an ability of cloud-based computing that simplifies sharing data. Storing by learners and accessing by an instructor are a basic requirement of digital tools for implementing formative assessment. For instance, learners use computers and connect to the Internet for doing and submitting an assignment. It can simplify many tasks about assignment procedure, such as the Google Spreadsheets can help an instructor to make questioning and answering easy. An instructor creates a sheet, writes questions, and then requests learners to answer on reserve locations. The AudioNote is available for uploading voices of answers. Answers in the form of shape, sketch, and annotation are available in the Evernote Skitch. The effectiveness of cloud-based computing is it is less time-consuming in gathering evidence task, but it cannot reduce the running time of assessing task. For developing formative assessment in a classroom situation, the automatic assessment is required to empower the suitable strategy.

### An automatic concept map assessment

The human-based assessment is one alternative of concept map assessment, but its major issue is it is time-consuming when there are many concept maps. Another alternative method to reduce being time-consuming is the automatic concept map assessment based on a computerized assessment. Several researchers proposed the designing and implementing software to support a construction of concept maps and developed automatic concept map assessment for using in their tasks (Luckie et al., 2004, 2011; Cline et al., 2010; Hirashima et al., 2011). A criteria map is the most popular tool to use in an automatic assessment that can influence the effective assessment. The criteria map is constructed by an expert and is used to control quality and quantity of propositions. The difference between handmade assessment and computerized assessment is flexibility because the computerized assessment requires the strict rules for calculating the concept map score. Although the handmade method is more flexible than the automatic method, the handmade method takes more time than computerized assessment.

For increasing the flexibility, some systems assign an additional condition of scoring methodologies such as graph theory, pattern of propositions, ranging scoring, or synonym words (Tsai et al., 2001; Hoeft et al., 2003; Kornilakis et al., 2004; Harrison et al., 2004; Anohina-Naumeca & Grundspenkis, 2009). It seems like the flexibility of handmade assessment, but the additional condition is defined depending on the objective. For example, an addition of graph theory disregards linking words for giving more score. The learners who construct the incorrect proposition can receive a partial score when two concepts have a relation or can be connected to each other, even though the linking word is incorrect.

### A comparison of automatic concept map assessment tools

Table 1 shows the systems that use the automatic concept map assessment and their criteria (Pailai et al., 2016a, b). In this table, we divide the group of criteria into three groups. The first group is component providing based on the criteria map which includes the label of concepts and the label of relations. A group symbol is represented as the superscript number. The additional components provided of C-TOOLS (Luckie et al., 2004, 2011) are distractor of concept labels or linking words, and blank cards. Also, the blank cards are the additional component of CMT (Cline et al., 2010), while Kit-Build (Hirashima et al., 2011) concept map provides only the label of concepts and label of relations. The provided components have a direct effect on the assessment method. That means the method should cover and complete all of the propositions which are possible in learner maps.

**Table 1:** The systems and assessment criteria

System	Criteria
C-TOOLS (Robograder)	C^a^, R^a^, D^a^, B^a^, P^b^, CM^b^, S^b^, I^c^
CMT (rule based)	C^a^, R^a^, D^a^, P^b^, CM^b^, S^b^, I^c^
Kit-Build concept map	C^a^, R^a^, P^b^, CM^b^, E^b^, I^c^, G^c^

*C* concepts with word/label, *R* connected link with a linking word, *D* distractor of concept labels/linking words, *B* blank cards, *P* propositions, *CM* criteria map, *S* synonym matching, *E* exact matching, *I* individual assessment, *G* group assessment

^a^Provided component

^b^Assessment

^c^Results

The second group is assessment methodology. The primary methodology of the assessment process is proposition level exact matching, which can identify correct and incorrect propositions clearly and can report the results immediately. An additional method is a synonym matching for measuring the label of the incorrect proposition. After using the proposition level exact matching, the incorrect proposition will be sent to the synonym finder such as WordNet (Kornilakis et al., 2004; Harrison et al., 2004). In this case, the synonym finder will annotate a value (density value) of label word of the incorrect proposition. So, the automatic assessment will generate a total score of the map that includes proposition level exact matching score and synonym matching score. The synonym matching is the additional assessment when the system provides the extra component such as blank cards to learners.

The last group is the results of the automatic assessment. These three systems can provide individual results between the criteria map and each learner map. Besides, an additional result is a group assessment, which includes a group map, a group-goal similarity score, and a group-goal difference map. It only occurs in the Kit-Build concept map. The automatic concept map assessment can inform the information of learners to the instructor in a short time, which can reduce the running time of assessment process immediately. However, the number of learners is still a problem when designing and providing the instructor’s feedback in a class period. To find the overview of class shortly, the group assessment can provide the information better than picking some individual results. Thus, the group assessment ability is an advantage of the Kit-Build concept map over the other automatic assessment systems when it is utilized in the environment with time limitation.

### An arrangement of the Kit-Build concept map

**Fig. 5:**
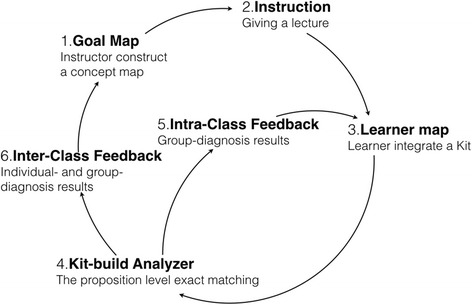
A cycle of the Kit-Build concept map on formative assessment

The arrangement of the Kit-Build concept map on formative assessment in a lecture class consists of six steps as shown in Fig. 5. The first step as the general scenario of the lecture class, an instructor creates lecture contents and then constructs a goal map for representing a learning goal of the class. The next step is giving the lecture to learners in a class period. During the lecture, the instructor can check the learners’ understanding by requesting learners to construct a learner map. Then, the diagnosis results are reported to the instructor immediately for informing about current understanding of learners. These steps are gathering and assessing the evidence of learners. The fifth step is providing intra-class feedback during the class period, which requires an instant practical information for capturing an overall understanding of class. This requirement is responded by the group-diagnosis results that include the group map which can inform the common understanding, and the group-goal difference map which can inform the common misunderstanding of class in one map. Finally, the inter-class feedback is information analysis of the previous class to improve the understanding of learners on next chance and to improve the lecturing of next classes. It is possible to use both individual- and group- diagnosis results for discovering the issue of the previous lecture.

The arrangement of the Kit-Build concept map on formative assessment is efficient flow to fulfill formative assessment cycle. The automatic concept map assessment can help the instructor to reduce the workload of an assessment process, and the diagnosis results can provide an opportunity of an instructor to improve understanding of learners immediately. Based on these abilities, the Kit-Build concept map can create a chance as much as possible to form and complete the formative assessment cycle (Pailai et al., 2016a, b). For answering the key questions of the formative requirement, a goal map is an answer of where learners’ questions are going. Gathering and assessing learner’s evidence in the form of concept maps can identify the current understanding of learners, which is an answer of where learners’ questions are now. The diagnosis results are the practical useful information that can contribute instructor’s feedback, which is an answer of how to close the gap question. Not only gathering the evidence of learning in the class period, Kit-Build concept map covers assessing the evidence for designing and providing the feedback of the instructor.

## Results

### The procedure of the practical use

The investigation will focus on the improvement of learners after they received instructor’s feedback. In a practice setting, we have a topic “See from northern hemisphere, the sun rises from the eastern sky, passes through the southern sky, and sets in the western sky” (Yoshida et al., 2013b). An instructor divided this topic into two sub-topics that include “the sun’s orbit seen from northern hemisphere” in the first practice and “the sun’s orbit seen from southern hemisphere” as an advanced topic in the second practice. The participants are learners in the third grade in elementary school, which contain two classrooms as group A and group B. The number of participants is 38 in each group, and the class period is 45 min for each group. The instructor requests learners to construct learner maps three times in each class, which learners have to construct each map in 5 min. The first map request happened in the middle of the class period. The first request is to identify the current understanding of learners after a lecture. Afterward, the second request is given after instructor provided the feedback as supplementary lecture to learners. So, the results of the second learner maps can report a progress of learners and shows an effectiveness of instructor’s feedback which is designed based on the diagnosis results. The last request is a chance to reassess the understanding of learners, and report the effectiveness of instructor’s feedback through the improvement of learners. In this context, the instructor already has the expectation on lecture contents before a class that is the learning goal of the class in the form of a goal map. To accomplish the learning goal, the instructor anticipates learners to have more progress at every checkpoint.

### An effectiveness of intra-class feedback

**Fig. 6:**
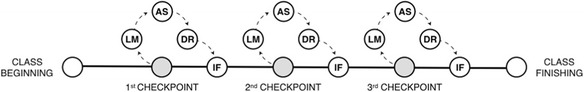
Practical flow of intra-class feedback in the lecture class

The practice is designed for assessing the effectiveness of intra-class feedback by repeated three times of an inner loop of the cycle (Fig. 5). Figure 6 illustrates the practical flow that is used in both groups. The first, second, and third checkpoint are gathering learner maps (LM) and assessing the learner maps (AS) by using Kit-Build concept map. The results of these processes are diagnosis results (DR), which are used to design instructor’s feedback (IF) and decide next actions of the instructor. We present the practice results to investigate the effectiveness of intra-class feedback that can be explained in more detail of each step in practical uses. From this section, the group-goal difference map will be shown only the lacking link for focusing on the mistakes of learners. And the improvement of learners is represented by decreasing the number of lacking links which also presents the effectiveness of instructor’s feedback together.

**Fig. 7:**
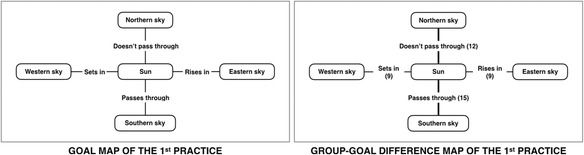
The goal map and the group-goal difference map of the first practice

In the lecture class of the first practical use, the instructor requests learners to construct learner maps in the middle of class. Figure 7 shows the goal map of “the sun’s orbit seen from northern hemisphere” and the diagnosis results in the form of the group-goal difference map at the first checkpoint of group A. The group-goal difference map reports the lacking links tagged with the number of learners who did not construct those propositions. It shows the weakness of learners on the lecture content. The maximum tagged number of each lacking link is equal to the number of learners of the class, so the total of maximum tagged number is the multiplying number between the number of learners and the number of goal map links. In this case, the group-goal difference map can identify critical areas that suggest to the instructor to focus at that time. The most different understanding of the first checkpoint is “pass through” link that is connected to the “Southern sky” concept and the “Sun” concept.

**Fig. 8:**
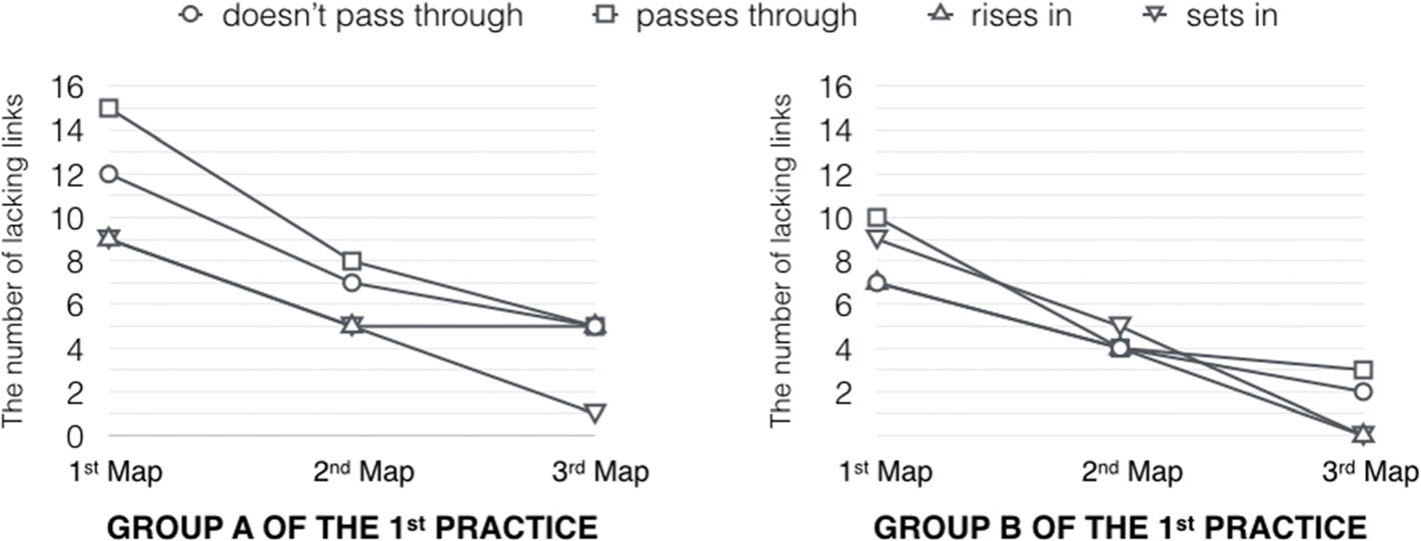
The number of lacking links of each group of the first practice

The diagnosis results point out the critical areas and suggest the instructor to analyze those areas based on the results of the proposition level exact matching methodology. Even though the instructor explained about the content which covers the related contents of those lacking links in lecturing, the instructor judged that the explanation was not clear enough. Accordingly, the instructor relocated the visualized lacking links of group-goal difference map for clear visibility and showed to learners directly when the instructor gave the feedback as supplementary lecture. Since gathering and assessing the learner’s evidence until providing the feedback of the instructor, these processes are implemented to fulfill a cycle of formative assessment in the lecture class. The improvement of learners is useful when implementing each formative assessment cycle. To complete another formative assessment cycle, the instructor requested the learners to reconstruct the second map and the third map for reassessing the understanding of learners after they received each instructor’s feedback, which is repeating of formative assessment cycle. Figure 8 shows the number of lacking links of each group. The practice results represent the decreasing number of each lacking link every time learners received the instructor’s feedback. The practice of intra-class feedback can demonstrate instantaneous assessment ability of the Kit-Build concept map which is the contribution to the implementation of formative assessment.

In this situation, the Kit-Build concept map generated the diagnosis results of each learner automatically that are the similarity score of each learner map and 38 individual-group difference maps. The instructor can recognize the current understanding of each learner individually based on those results, which need to take a long time for analyzing all of them. The time limitation is the most significant problem of a lecture class. Although automatic concept map assessment can reduce time consumption of assessing learner maps, the number of learner maps is still a problem when instructor analyzes the individual-diagnosis results. This problem means it is hard to recognize all of the individual-diagnosis results on the class period such as 38 results in one class period. Thus, the valuable information of the Kit-Build concept map is group-diagnosis results which are practical information on a class period. The group map presents common understanding, while common misunderstanding is presented in form of the group-goal difference map. Especially, the group-goal difference map where the instructor can use to recognize the most common misunderstanding of learners as the first priority for helping the learners. The number of each lacking link indicates the number of learners who struggle on the propositions, and who need help from the instructor to raise their understanding.

The diagnosis results of the first checkpoint present the effectiveness of the lecture. As instructor’s expectation on learners, learner maps should be same with the goal map that can reveal learners’ understanding about the lecture content well. This situation is a positive lecture of the classroom situation. However, the practice results present that learners cannot follow all of the instructor’s expectation at the first checkpoint. The group-goal difference map of the first checkpoint of group A is illustrated on the right-hand side of Fig. 7. The lacking links are used to indicate the misunderstanding of learners which the degree of misunderstanding is indicated by the indicator that includes tagged number and the weight of line. There are four possible lacking links based on the goal map (the left-hand side of Fig. 7) before the instructor was informed of the group-goal difference map. Even all of four links are possible to appear on the diagnosis results, the diagnosis results can suggest which the most important lacking link is. Therefore, the instructor focused on the highest tagged number of the lacking links on the group-goal difference map that becomes the first priority for solving at the time (Sugihara et al., 2012). In other words, the information of the diagnosis results can indicate evidently the misunderstanding of learners for confirming or redirecting the supplementary lecture of the instructor. Accordingly, the diagnosis results can contribute the informative feedback and can encourage the effective action of the instructor.

In the first map of group A, the total number of lacking links is 45 links as shown on the right-hand side of Fig. 7, which is equal to 29.61 percentages of all possible lacking links (152 links from 4 links of each 38 learner maps). Moreover, the diagnosis results suggest an important link that is the most number of lacking link. So, the “pass through” link causes the most misunderstanding among learners (15 learners from 38 learners of the class), and the instructor took the link as the main content of feedback in the form of the supplementary lecture. Subsequently, the instructor gave the feedback that emphasized on the “pass through” link especially more than the other lacking links. A line graph on the left-hand side of Fig. 8 represents the effectiveness of the feedback. The line graph of group A shows the decreasing of lacking links of all three maps. In this context, the number of lacking links at the “second map” was decreased when compared with the lacking links of the “first map” that means the learner’s understanding was increased after the instructor gave the feedback to learners. The total number of lacking links at the second map of group A remained 25 links that were decreased 55.56 percentages from the first map, and the lacking links of this second map are equal to 16.45 percentages of all possible lacking links. Also, the diagnosis results of the second checkpoint of group A suggest that the “pass through” link still the most number of lacking links, although the “pass through” link is the most decreased link among the lacking links from the first map. Another candidate link is the “does not pass through” link (7 tagged number), which the number of the link is not too much different from the “pass through” link (8 tagged number). So, the instructor designed the second feedback of group A based on these lacking links. Finally, the lacking links of the third map are presented in the line graph on the left-hand side of Fig. 8 as the “third map”. The total number of the lacking links is 16 links that means in the third map remained only 10.53 percentages of all possible lacking links.

Afterward, the instructor conducted the second class on the same topic with the same instructional plan for investigating the effectiveness of intra-class feedback. The line graph on the right-hand side of Fig. 8 represents the number of each lacking link in every map of group B. The diagnosis results of the first checkpoint of group B identify that the “pass through” link is the most misunderstood, which is the same to that most misunderstood of the previous class (group A). So, the instructor gave the intra-class feedback by using the “pass through” link as the main content of supplementary lecture before the instructor requesting learners to construct the map again. Subsequently, the number of lacking links of the second checkpoint is shown at the “second map” of the right-hand side of Fig. 8. The most lacking link is not the “pass through” link, but it changed to the “sets in” link that means the feedback can help the learners to understand the content of the “pass through” link. However, the situation of group B was different from group A. From the suggestion of the diagnosis results, the “sets in” link became the most number of lacking links instead of the “pass through” link. Then, the instructor changed the main content of supplementary lecture to the “sets in” link following the current learning situation. Next, the third checkpoint of group B presents the number of lacking links at the “third map” on the right-hand side of Fig. 8. The “sets in” links were indicated as the most misunderstood of the second checkpoint that disappeared in the third checkpoint after the instructor took the link as the main content of the feedback. Hence, the emphasis of the instructor on “sets in” in the second feedback can remove the “sets in” link from lacking links of the third checkpoint directly.

Accordingly, the first practical use of the Kit-Build concept map can illustrate the ability of the Kit-Build concept map that is adequate technology-enhanced learning for implementing and facilitating the learning environment of formative assessment. It was used to complete three cycles of formative assessment in the lecture class, and the results of practical use demonstrated the effectiveness of intra-class feedback when the instructor received the current learning information in the form of the diagnosis results.

In addition, we have the comparison between learner map score and standard test score, and we produced mini-test about the same topic in each practice. The standard test of science learning is the National Japanese Exam (NJE), in which the content is general science domain. And the mini-test is a quiz at the end of the topic that examines in the same topic with the lecture topic of the practical uses. The learner map score is the ratio of the number of correct propositions in learner map to the number of propositions in the goal map. It presents the degree of accordance between the learner map and the goal map that takes a value of 0 to 1. The correlation coefficients between the third map score and standard test of science learning, and the correlation coefficients between the third map score and mini-test score are contained in Table 2. The average of the third map score in group A is 0.882 (SD = 0.285) that have the correlation coefficient with a standard assessment of science which is 0.337. The result is statistically significant (*N* = 38, *p* = 0.039). Also, the correlation coefficient between the average third map score and mini-test is 0.395. The result is statistically significant (*N* = 38, *p* = 0.014). These results suggest the quality of learner map would reflect the understanding of learners on the lecture content. In contrast, the correlation coefficient in group B is low because of ceiling effect of some learners. The average of the third map score in group B is 0.967 (SD = 0.117). The summation between the average score of the third map and the standard deviation is higher than the maximum value of the learner map, which is statistically confirmed by the ceiling effect. The results represent the inter-class feedback of the instructor can improve learning achievements in the lecture class when utilizing the Kit-Build concept map on formative assessment.

**Table 2:** Correlation coefficients in the first practice

	Group A	Group B
Standard test of science learning^a^	0.337 (*p = 0.039*)	−0.170 (*p = 0.307*)
Mini-test^b^	0.395 (*p = 0.014*)	0.284 (*p = 0.081*)

^a^The National Japanese Exam (NJE)

^b^The quiz at the end of the topic

### An effectiveness of inter-class feedback

**Fig. 9:**
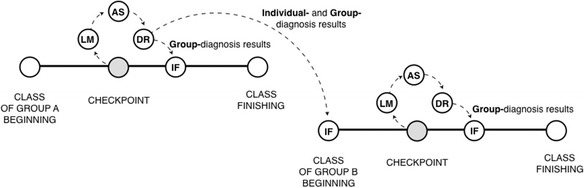
Intra-class feedback and inter-class feedback in the lecture class

Following the first practice that explains the contribution of the Kit-Build concept map on intra-class feedback, the group-diagnosis results can identify the critical areas, and encourage the instructor to produce proper feedback. And the intra-class feedback can help learners to achieve the learning goal of class in the class period immediately. In the second practice, we present another classroom situation that the intra-class feedback cannot improve learning achievements immediately. The practice flow is designed for assessing the effectiveness of intra-class feedback and inter-class feedback by repeating both the inner and outer loop of the cycle (Fig. 5). The second practice setting requests learners to construct learner maps three times, and the instructor provides the feedback every time after he/she got the diagnosis results the same as the previous practice. Also, the lecture content relates to “the sun’s orbit seen from southern hemisphere,” which is an advanced topic of the previous practice. The class period of the second practice is 45 min, and the learners have to construct each learner map in 5 min, which the first map request happened in the middle of the class period. Figure 9 illustrates practice flow of intra-class feedback and inter-class feedback in the lecture class.

**Fig. 10:**
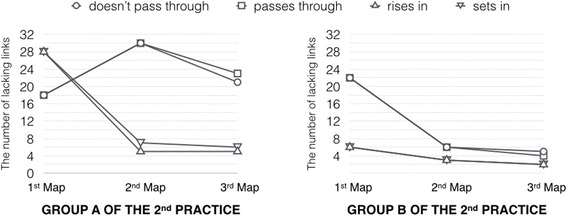
The number of lacking links of each group of the second practice

The instructor received the diagnosis results that are the information of current learning situation in the class. The first diagnosis results of group A is presented at the “first map” in the line graph on the left-hand side of Fig. 10. The information of the diagnosis results suggests that the most number of lacking links consists of the “rises in” link and the “sets in” link. Thus, the instructor emphasized the lecture content of these links for improving learner’s understanding. The main content of intra-class feedback based on the current learning situation as the “rises in” link and the “sets in” link is emphasized more than the two other links. Afterward, the instructor requested his/her learners to construct the learner maps again for reassessing the learning situation after they had been given the intra-class feedback, which is the same activity when using the Kit-Build concept map in the lecture class and also started the new cycle of formative assessment. The “second map” on the left-hand side graph of Fig. 10 shows the number of lacking links of the second checkpoint that represents the effectiveness of the intra-class feedback, which the instructor emphasized on the lecture content of the “rises in” link and the “sets in” link intentionally. The line graph illustrates the decreasing of the lacking links which are the main content of supplementary lecture following is the “rises in” link which was decreased by 82.14 percentages and the “sets in” link was decreased by 75.00 percentages from each its number of lacking links of the first checkpoint.

However, the negative situation happened in group A of this second practice because all of the lacking links should be decreased after the learners received the intra-class feedback as the situation of the first practice. There is the increasing of lacking links that include the “does not pass through” link and the “passes through” link. In this situation, the learners have more understanding about the “rises in” link and the “sets in” link because they had been given the supplementary lecture on these related lecture content. So, they can construct the correct propositions on the second learner maps more accurately. On the other hand, the reconstructing of the learner maps effected to the other links and the learners still have the confusion about the “does not pass through” link and the “passes through” link. Hence, the instructor tried to emphasize on the related lecture content of the most number of lacking link again. The main content of the second intra-class feedback was changed from the “rises in” link and the “sets in” link to the “does not pass through” link and the “passes through” link based on the active information of the diagnosis results. Finally, the “third map” in the line graph of the left-hand side of Fig. 10 presents the number of lacking links after the instructor gave the second intra-class feedback of group A. The results indicate the number of lacking on the “does not pass through” link and the “passes through” link still higher than the first checkpoint. There is no more chance for gathering and assessing learner’s evidence because the time of class period is running out. Thus, these lacking links requested the instructor to analyze them after over the class when the instructor has more time to analyze the issue of the previous class.

Subsequently, the instructor investigated the information of group A for finding and solving the ineffectiveness of lecturing and intra-class feedback. The diagnosis results of group A identify that the intra-class feedback can improve the understanding on the “rises in” link and the “sets in” link. However, there is the confusion between the “does not pass through” link and the “passes through” link which cannot improve the understanding by using only supplementary lecture. The analysis results of the instructor are the following: (1) the lecture topic of “the sun’s orbit seen from southern hemisphere” is an advanced topic of “the sun’s orbit seen from northern hemisphere.” The instructor judged that the lecture content was more difficult for learners than instructor’s expectation and the problem is the difficulty in thinking in which direction in the sky that the sun can be seen. (2) Based on the confusion between the “does not pass through” link and the “passes through” link, the instructor found that the relative position was not indicated in the lecture content of group A. (3) The results of group A represent ineffectiveness of intra-class feedback on the “does not pass through” link and the “passes through” link, so it is necessary to adjust the instructions plan by using supplementary material that includes terrestrial globes, lights, and small dolls. Thus, the inter-class feedback is the adjusted instructional plan for referring to the relative position and the enhancement lecturing by using the supplementary material. Also, the instructor expected the inter-class feedback could help the learners to understand the lecture content more than the previous class.

Afterward, the lecturing of group B was conducted following the adjusted instructional plan which the effectiveness of lecturing is presented at the “first map” on the right-hand side graph of Fig. 10. The number of lacking links is less than the previous class on the same checkpoint. The lacking links have the characteristic as the instructor expectation: (1) the “rises in” link and the “sets in” link are possible to decrease by adjusted the lecture content as the supplementary lecture of group A. (2) The learners of group B were also confused on the “does not pass through” link and the “passes through” link which is the same situation of group A. So, the intra-class feedback of group B was not only given the supplementary lecture but using the supplementary material for improving the learner’s understanding which the results of the second learner map can demonstrate the effectiveness of these approaches. The number of lacking links of the second checkpoint is shown at the “second map” of the right-hand side graph of Fig. 10. The line graph illustrates the decreasing of the “does not pass through” link and the “passes through” link obviously. The number of lacking links of the second checkpoint of group B was decreased by 67.86 percentages from the first checkpoint, which is the effectiveness of inter-class feedback in the form of intra-class feedback. The adjusted instructional plan and the supplementary material can improve learning achievements since the first checkpoint of group B. Based on the information of the previous class and instructor’s experiences, the additional materials can improve the achievements of group B immediately. Moreover, the effectiveness of intra-class feedback was turned into positive in group B. In this context, the results mention to the issue of the previous class which is the instructional plan, which is insufficient to explain the meaning of lecture content. The average of the third checkpoint score was 0.914 (SD = 0.201). Also, the correlation coefficient between the average score and standard assessment test score was 0.391 (*p* = 0.015) that is significant.

### Continuous effectiveness improvements

**Fig. 11:**
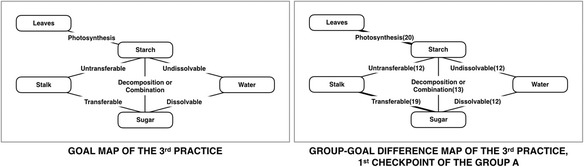
The goal map and the group-goal difference map of the third practice

In the previous practices, the first practice results display the effectiveness of intra-class feedback, and the second practice results show the effectiveness of inter-class feedback when intra-class feedback is insufficient to improve the understanding of learners. Finally, the third practice is designed for displaying the continuous effectiveness when both of intra-class feedback and inter-class feedback are effective for improving learning achievements in the lecture class. The third practice has two groups from the sixth grade that contain 36 subjects in group A and 40 subjects in group B. An instructor requested learners to construct learner maps two times in each group, and the topic of both groups is “decomposition of starch made by photosynthesis in leaves into sugar, and transfer to water-melted sugar through stalk” (Yoshida et al., 2013a). The class period of the third practice is 45 min, the learners have to construct each learner map in 10 min, and the first map request happened in the middle of the class period. The goal map contains five concepts and six relations with a linking word which are six propositions in a map. Figure 11 shows a goal map that is used in both groups and the first group-goal difference map of group A.

**Fig. 12:**
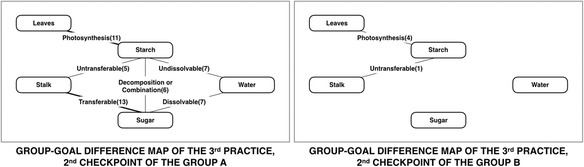
The group-goal difference map of the third practice

The group-diagnosis results of the first checkpoint of group A display that the “Photosynthesis” link is the most common current misunderstanding of learners, which instructor should pay particular attention to this link more than the other lacking links. The instructor emphasized on “Photosynthesis” link and focused on the information about the “Leaves” concept and the “Starch” concept. The instructor made supplementary lecturing as intra-class feedback based on the suggestion of the diagnosis results for improving the understanding of learners on critical areas. Subsequently, the instructor provided the feedback to the learners and requested learners to reconstruct a map again. Then the results of intra-class feedback present the number of “Photosynthesis” link decreased to less than the “Transferable” link, which is illustrated in Fig. 12. Accordingly, the instructor already has individual- and group-diagnosis results which are the previous class information when finishing the practice of group A. It can help the instructor to adjust and improve their instructional plan. Especially, the instructor already knows the way to improve on learners’ understanding based on the information of the previous class.

Table 3 shows the percentage of average score that includes science test of science learning score, the first checkpoint score, and the second checkpoint score. The average score increases 17.7 percentages and responds to the number of lacking links which decreases more than 50 percentages. Moreover, the instructor improved the instructional plan for group B based on the information of group A in order to emphasize the links over the instructor’s expectation of group A. The results show an average score of the first checkpoint in group B is more than the average score of the second checkpoint in group A. The results demonstrate the effectiveness of both intra-class feedback and the inter-class feedback, which contributes the higher average score in group B. The average score of the second checkpoint of group B increases 18.7 percentages that respond to the number of lacking links, which decreases more than 90 percentages.

**Table 3:** Percentage of average score

Average score	Group A	Group B
Standard test of science learning^a^	63.2	63.6
1^st^ checkpoint	61.4	79.2
2^nd^ checkpoint	79.1	97.9

^a^The National Japanese Exam (NJE)

## Discussions

### The advantage of the Kit-Build concept map

Educational enhancement through technology can help to improve learning achievements. An instructor remains to be the most influential of the class who cooperate and select the learning strategy in the instructional plan. The Kit-Build concept map is a digital tool for supporting concept map strategy, which is instantaneously available on a wide variety of scenario in class. Correspondingly, the practice results have illustrated that the ability of the Kit-Build concept map can arrange on formative assessment to fulfill the cycle as more as possible. The details of formative assessment might be different that depends on the instructor, although the Kit-Build concept map has adequate ability to contribute to the gathering and assessing of the evidence of learners and encourage the instructor to develop a positive classroom situation. The concept map strategy is used to create the learning goal of class and to elicit the understanding of learners. The goal map and the learner maps can be used to confirm the current understanding between the instructor and the learners on the same lecture content that represents in the form of the diagnosis results. Exclusively, the diagnosis results of learner’s evidence (individual-diagnosis results) and additional evidence of learners (group-diagnosis results) are practical information on the contribution of instructor’s feedback designing of both intra-class and inter-class feedback. Accordingly, the classroom environment of the Kit-Build concept map can provide opportunities to close the gap between current and desired performance, and also provides information to the instructors that can be used to shape the lecturing. These are principles of good feedback practice (Nicol & Macfarlane-Dick, 2006).

### The valuable information in lecture class

The class period is time limitation of a lecture class, which the instructor can control his/her class following the preparation of the class as an instructional plan in general situation. Also, the instructional plan includes the expectation and prediction of the learners based on the instructor’s experience in managing the positive and negative situation on the class. The positive case is an ideal situation such as all of the learners can understand well on the lecture content, which the learning achievements are represented through the test score or map score. Another situation is the negative case such as unexpected situation. Accordingly, the instructor can select the ways to duel with the immediate situation based on the preparation and his/her experience as the prompt immediate feedback to the learners. The importance of providing immediate feedback is beneficial for learning achievements and motivation (Narciss & Huth, 2006; Draper, 2009; Li et al., 2010). However, observing evidence of the situation and identifying the problem are the most important task of deciding the effective actions. The learning evidence can identify the current learning situation obviously whatever positive- and negative-situation in the class, which is the information for contributing the effective actions of the instructor.

The Kit-Build concept map takes action as an assistance to duel with time limitation, which facilitates learners to create learning evidence in a class period and also identify the current learning situation on time. Subsequently, the instructors can observe the information via the diagnosis results immediately. The expected situation was presented in the first practice, and the instructors can improve learning achievements by intra-class feedback because the instructors can address the critical problem of the class and then give the supplementary lecture on the problem to elevate the learner’s understanding. Also, the second practice represents the unexpected situation which cannot solve in the class period immediately. The ineffectiveness of the intra-class feedback was showed as the unexpected situation of the class. Eventually, the problem was solved in the next class in the form of the inter-class feedback based on the learning evidence of the previous class. The supplementary material was used to enhance lecturing and the learning achievements were increased.

### Stakeholders feedback

The practices emphasize on encouraging learning in a lecture class and supporting the instructor who wants to share knowledge to learners. The instructor anticipates learners to understand lecture content following the instructor’s expectation. Misunderstanding of learners is an undesirable situation that often appears in classroom situations. Correcting the misunderstanding is the simple way for improving learner’s understanding, but it is difficult to find the critical areas, which is the misunderstanding of learners on the lecture contents. Correspondingly, the diagnosis results of the proposition level exact matching methodology are a crucial ability of the Kit-Build concept map to identify the critical areas quickly and obviously. The diagnosis results can address exact critical parts of the contents that the learners make mistakes in and the instructor could not think about those parts before, which is considered to be useful information. These are positive opinions from the instructors who used the Kit-Build concept map in the practices. In addition, we conducted a questionnaire survey about the usefulness of the Kit-Build concept map from learners’ aspect when used in the classroom situation. The questionnaire survey consists of nine questions in a 5-point scale. And the learners are the participant in the first practice and the second practice. Accordingly, we gained totally positive opinions from learners such as “It was fun to make maps” and “It was easy to make a map.” It can present the usefulness and usability of the Kit-Build concept map when using in the lecture class from the learners’ aspects.

## Conclusion

The Kit-Build concept map is a digital tool for creating the learning environment to improve learning achievements, especially the formative assessment in lecture class which is reported in the form of practical uses when used in elementary school. The evidence-based feedback of an instructor is a key of formative assessment to improve learning achievements in the classroom situations. The contribution of the Kit-Build concept map is the ability for cooperating with the instructor to implement the formative assessment via a concept map strategy, facilitating the learning process in the form of a digital tool, and creating an opportunity to improve learning achievements in the classroom situation. The ability of Kit-Build can create a chance for completing the formative assessment cycle as more as possible and saving the time of instructor and learners. Hence, gathering, assessing, and providing the information of current learning situation are the crucial contributions on formative assessment of the Kit-Build concept map. The kit and the proposition level exact matching methodology are used to confirm the understanding between instructor and learners on the lecture content. Also, the diagnosis results can identify the propositions which require supplementary lecture for filling on lacking understanding of learners. Lastly, the results of the practices can describe the effectiveness of the formative assessment when the Kit-Build is utilized in the lecture class. It can illustrate that Kit-Build concept map is a suitable digital tool for applying on formative assessment in a lecture class.
